# Antifungal Activity of the Ethanol Extract from* Flos Rosae Chinensis* with Activity against Fluconazole-Resistant Clinical* Candida*

**DOI:** 10.1155/2017/4780746

**Published:** 2017-02-20

**Authors:** Lulu Zhang, Hui Lin, Wei Liu, Baodi Dai, Lan Yan, YongBing Cao, Yuan-Ying Jiang

**Affiliations:** New Drug Research and Development Center, School of Pharmacy, Second Military Medical University, Shanghai 200433, China

## Abstract

This study was designed to investigate the antifungal activity of a hydroalcoholic extract from* Flos Rosae Chinensis* (FRC) combined with fluconazole (FCZ) against clinical isolates of* Candida albicans* resistant to FCZ. The minimum inhibitory concentration (MIC) of FRC was determined using a checkerboard microdilution assay. The synergistic effects of the combination of FRC and FCZ against clinical isolates of* C. albicans* resistant to FCZ were further confirmed by constructing time-growth curves and performing an agar diffusion test. FRC alone exerted efficient antifungal activities against* C. albicans* within a MIC_80_ ranging from 20 *μ*g/ml to 40 *μ*g/ml. FRC failed to enhance the effects of FCZ against sensitive* C. albicans* strains, although it rendered FCZ-resistant* C. albicans* more sensitive. These results were further confirmed by the result of in vivo study. Our study is the first to discover that FRC can inhibit the growth of* C. albicans* to a certain degree. An FRC antifungal mechanism study showed that FRC strengthens FCZ to inhibit the action of ergosterol biosynthesis by promoting the transformation of lanosterol to eburicol, suggesting that the antifungal mechanism of FRC involves the inhibition of ergosterol biosynthesis.

## 1. Introduction

In the modern society, with the increasing use of cancer chemotherapy, organ transplantation, and hematopathy and the increased incidence of diabetes and diseases of aging, broad-spectrum antibiotics, adrenal cortical hormone, cytotoxic drugs, and immunosuppressants have been clinically applied in an unreasonable manner for a long time. The morbidity and mortality of fungal infections (mainly caused by* C. albicans*) have been on the rise [1–3]. FCZ is the most widely used azole drug in clinical settings. With the increasing use of FCZ, drug-resistant strains are emerging rapidly [4–6]. How to exploit new antifungal drugs or to identify non-resistance-forming methods of killing* C*.* albicans* remains one of the hottest issues in antifungal research. It is known that a variety of Chinese herbal medicines exert activities against pathogenic microorganisms [7–11]. Moreover, antifungal activities of extracts from* Morus mesozygia* [12],* Toddalia asiatica* [13],* Wrightia tinctoria* [14],* Vismia rubescens *[15], and many other herbs have also been reported by other labs. Studies at our research center have shown that baicalein [16], berberine [17], and tetrandrine [18] could enhance the antifungal activity of FCZ against FCZ-resistant* C. albicans*. Our study was conducted to identify new compounds from Chinese herbal medicines that can synergistically potentiate the inhibitory effects of FCZ on the growth of* C. albicans*.

## 2. Materials and Methods

### 2.1. Ethics Statement

All mice were obtained from SLAC Laboratory Animal Co. Ltd., Shanghai, China. They were housed under controlled temperature (23 to 25°C) and lighting (8:00 a.m. to 8:00 p.m. light, 8:00 p.m. to 8:00 a.m. dark) and with free access to standard food and drinking water. All animal experiments were approved by the Administrative Committee of Experimental Animal Care and Use of the Second Military Medical University and were performed strictly in accordance with the National Institutes of Health guidelines on the ethical use of animals. All surgery was performed under sodium pentobarbital anesthesia, and all efforts were made to minimize suffering.

### 2.2. Plant Material Extraction

*Flos Rosae Chinensis* as a commonly used Chinese traditional medicine is the dry flower of* Rosa chinensis* Jacq. in Rosaceae. It was bought from having drug supply certificate pharmacy. Approximately 5 g of dried and smashed crude drug was extracted two times with an excess 100 ml of 70% ethanol, after which the extract was filtered. After removal of the solvent by rotary evaporation under reduced pressure at 50°C, the residue was dissolved with distilled water to approximately 0.5 g/ml FRC. So the amount of 10 mg/ml of FRC is equivalent to 1 g of crude drug. The FRC mentioned in the article refers to the hydroalcoholic extract. The final extract was dark brown liquid and stored at 4°C for further use.

### 2.3. Strains and Media

Thirteen clinical isolates of FCZ-resistant* C. albicans*, three clinical isolates of FCZ-sensitive* C. albicans*, one international calibration strain (SC5314), two ATCC-typed* Candida* strains (*C. albicans* ATCC10231 and* C. parapsilosis* ATCC90018),* C. parapsilosis* 160,* C. krusei* 4996, and* C. tropicalis* 2718 were utilized. All clinical isolates and other fungi were provided by the Shanghai Hospital (Shanghai, China) and their drug resistance was identified by the hospital clinical laboratory microbial group. SC5314 was kindly provided by William A. Fonzi (Department of Microbiology and Immunology, Georgetown University, Washington, DC). Strains were cultured at 30°C under constant shaking (200 rpm) in complete liquid medium (yeast extract peptone dextrose—YPD) consisting of 1% (w/v) yeast extract, 2% (w/v) peptone, and 2% (w/v) dextrose.

### 2.4. Antifungal Susceptibility Test

An antifungal susceptibility test was performed on all strains according to CLSI (formerly NCCLS) methods (M27-A) [19, 20].* C. parapsilosis* ATCC22019 was considered a quality control strain and was tested in each assay. The final concentration of fungus suspended in RPMI 1640 medium was 10^3^ colony-forming units (CFU)/ml, and the final concentration ranged from 0.125 to 64 *μ*g/ml for FCZ and 10^4^ to 19.53 *μ*g/ml for FRC. The* Candida* plates were incubated at 35°C for 24 h. Optical density (OD) was measured at 630 nm, and the background OD was subtracted from that of each well. Each strain was tested in triplicate. MIC_80_ refers to the concentration at which 80% of the tested strain was unable to grow. The fractional inhibitory concentration (FIC) index was defined as the sum of the MIC_80_ of each drug when the drug used in combination was divided by the MIC_80_ of the drug used alone. Synergy and antagonism were defined by FIC indices of ≤0.5 and >4, respectively. An FIC index result of >0.5, but ≤4 was considered indifferent [21].

### 2.5. Agar Disk Diffusion Assay

*C. albicans* 103 (an FCZ-resistant isolate with an MIC_80_ of 19.53 *μ*g/ml for FRC) was further tested by performing an agar diffusion assay [17]. A 100 *μ*l aliquot of 10^6^ CFU/ml suspension was spread uniformly onto YPD agar plates with or without 64 *μ*g/ml FCZ. Then, 6 mm paper disks impregnated with FRC and FCZ alone or in combination were placed onto the agar surface. Control disks contained 5 *μ*l of saline. Inhibition zones were measured after incubation at 35°C for 48 h. Assays were performed in duplicate.

### 2.6. Time-Kill Curve Studies

*C. albicans *103 in RPMI 1640 medium was prepared at a starting inoculum of 10^3^ CFU/ml [22]. FRC and FCZ were added at respective concentrations of 10 mg/ml and 10 *μ*g/ml for (in vivo achievable concentration of FCZ) [23]. At predetermined time points of 0, 12, 24, 36, and 48 h after incubation with agitation at 35°C, a 100 *μ*l cell suspension was withdrawn from every solution and serially diluted 10-fold in sterile water. A 100 *μ*l aliquot from each dilution was then streaked on a Sabouraud dextrose agar plate. Colony counts were determined after incubation for 48 h at 35°C. The experiment was performed in triplicate. Synergism and antagonism were defined as a respective increase or decrease of ≥2 log_10_ CFU/ml in antifungal activity produced by the combination compared with that of the more active agent alone, while a change of <2 log_10 _CFU/ml was considered indifferent [24].

### 2.7. Infection of Mice with* C. albicans*

Sixty ICR mice weighing 18–20 g (4–6 weeks old) were used once in the study. The experimental procedures were performed strictly in accordance with generally accepted international rules and regulations. ICR mice were equally divided into four groups (control, FCZ at 0.5 mg/kg, FRC at 0.4 g/kg, and a combination of FCZ at 0.5 mg/kg and FRC at 0.4 g/kg) to evaluate the synergistic effects of the combination of FRC + FCZ against FCZ-resistant* C. albicans* 103. Each group contained 15 mice. Immunosuppression was induced by intraperitoneal treatment with 80 mg/kg cyclophosphamide (Shionogi, Osaka, Japan) 3 days before infection [25]. Then, 1 × 10^5^ cells were inoculated in the mouse lateral tail vein. Two hours after fungal injection, the mice in the drug groups were treated with drugs in the amount of 0.2 ml/10 g of body weight [26]. Control mice were given the same volume of saline solution. Drugs were administered by gavage in liquid form once per day for 4 days. From grouping until the end of the experiment, the survival situations of mice were observed at a fixed time every day. The number of dead mice was recorded every day after treatment was given until all mice died.

### 2.8. Tissue Burden Assay

The kidney is the most frequent target of* C. albicans*, so the kidney burden assay is the most commonly used method to investigate infection with* C. albicans*. [27–30] Forty additional ICR mice were equally divided into four groups (control, FCZ at 0.5 mg/kg, FRC at 0.4 g/kg, and a combination of FCZ at 0.25 mg/kg and FRC at 0.2 g/kg) to evaluate the fungal burden in the kidney. First, 5 × 10^4^ cells were inoculated in the mouse lateral tail vein. The other operations were the same as those performed for the survival experiment. After 4 days of treatment, mice were euthanized immediately by sodium pentobarbital inhalation and processed for necropsy; the kidneys were excised via a sterile technique, weighed, and homogenized in 2 ml of sterile 0.9% saline. The homogenates were diluted 10-fold in sterile saline solution, and then 0.1 ml of each dilution and the undiluted homogenate were cultured in triplicate on Sabouraud dextrose agar (SDA) plates. Culture plates were incubated at 30°C for 48 h, and the number of CFU/g of tissue was calculated.

### 2.9. Sterol Analysis

*C. albicans* 103 was cultured overnight in YPD medium at 30°C with constant shaking (200 rpm). After 16 h of incubation, 2 ml from this suspension was subcultured for 24 h in 98 ml of YPD medium containing 0, 1 *μ*g/ml FCZ, 1 *μ*g/ml FCZ + 25 *μ*g/ml FRC, 1 *μ*g/ml FCZ + 250 *μ*g/ml FRC, 1 *μ*g/ml FCZ + 2500 *μ*g/ml FRC, or 2500 *μ*g/ml FRC. Cells were then washed three times and centrifuged. Then, 0.5 g of wet cells was weighed from each group, and 6 ml of 15% NaOH in 90% (V/V) ethanol and 2.5 ml of PBS buffer were added. The samples were heated at 80°C for 1 h. Nonsaponifiable lipids were extracted three times with 6 ml of mineral ether (30–60°C boiling) each time, and extracts were washed with 6 ml of sterile water. The washed extracts were evaporated to dryness at 60°C. The samples were dissolved in 0.5 ml of cyclohexane and stored at −20°C prior to analysis by gas chromatography-mass spectrometry (GC-MS). The operating condition used for GC/MS were as described by Zhang et al. [31].

### 2.10. Cytotoxic Assay

The capacity of FRC to inhibit cell growth was determined in vitro in human umbilical vein endothelial cells (HUVECs). The cytotoxic assay was performed as Chiesi et al. [32] The cells, cultured in Dulbecco's Modified Eagle's Medium (supplemented with 10% fetal calf serum, 2% penicillin, and streptomycin) in an incubator at 37°C under 5% CO_2_, were redistributed into 96-well microtiter plates at 8000 cells/well. After 24 h incubation, solutions containing FRC and FCZ were added to obtain final concentrations ranging from 160 *μ*g/ml to 0.625 *μ*g/ml and 32 *μ*g/ml to 0.125 *μ*g/ml, respectively. After 48 h of incubation, the solutions were discarded. Cells were incubated in 100 *μ*l MTT solution (90 *μ*l of DMEM + 10 *μ*l 3-(4,5-dimethylthiazol-2-yl)-2,5-diphenyltetrazolium bromide (MTT)) for an additional 4 hours. At this time, the succinodehydrogenase in the mitochondria of living cells can reduce the MTT to a water-insoluble blue-violet crystalline formazan and deposit in the cells, whereas dead cells do not. Then the MTT solution was aspirated carefully; 100 *μ*l of dimethyl sulfoxide (DMSO) solution was added to dissolve the formazan. After shaking for 10 minutes in the dark, the absorbance value was measured at OD 570 nm/630 nm, which can indirectly reflect the living cell number. The assay was performed in triplicate. LD_50_ refers to the concentration at which 50% of the tested cells died.

## 3. Results

### 3.1. FRC Inhibits the Growth of Part Fungi

The results of the checkerboard test are summarized in the tables. The FCZ-FRC combination markedly reduced the MIC_80_ required for FCZ-resistant* C. albicans* (Table 1). Synergism (100%) was observed for all 13 isolates of FCZ-resistant* C. albicans*. The corresponding mean FIC index was 0.165 (range 0.016–0.312). Indifference (100%) was observed for 5 isolates of FCZ-sensitive* C. albicans* (Table 2). In addition to* C. albicans*, synergism was observed for* C. parapsilosis* 160. Indifference was also observed for* C. krusei *4996,* C. tropicalis* 2718, and* C. parapsilosis* 90018 (Table 3). FRC inhibited the growth of 3 isolates of FCZ-resistant* C. albicans* in combination with other azole drugs. Indifference was observed for 3 isolates of FCZ-resistant* C. albicans* when FRC was combined with flucytosine or amphotericin (Table 4). Regardless of the MIC_80_ endpoints, antagonism was not observed in the combination group. In addition, FRC alone was efficient against* C. albicans* within an MIC_80_ range of 20–40 *μ*g/ml. From these results, FRC can inhibit the growth of most fungi [33].

**Figure 1:**
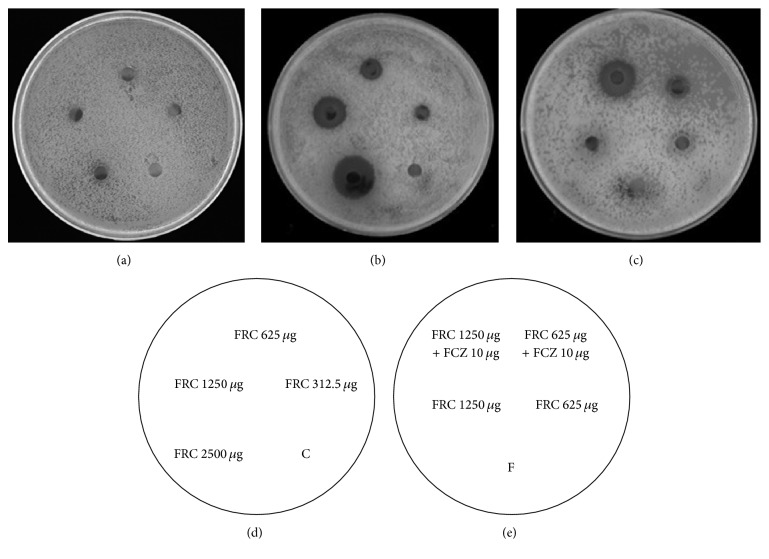
Agar disk diffusion assay to visualize the synergism of FCZ with FRC against clinical FCZ-resistant* C. albicans* isolate 103. (a) and (c) are plain agar plates; (b) is an agar plate containing 64 *μ*g/ml FCZ. (a) and (b) according to the description of (d); (c) as indicated in (e). C and F in the circle are controls.

Agar diffusion test can facilitate the visualization of synergistic interactions. FRC exerted no antifungal activity at any concentration (Figure 1(a)) and FCZ at 10 *μ*g showed only weak inhibition (Figure 1(c)). In contrast, on the agar plate containing 64 *μ*g/ml FCZ, FRC demonstrated a powerful antifungal effect (Figure 1(b)). The mean diameters of the inhibitory zones for 312.5, 625, 1250, and 2500 *μ*g FRC increased to 6, 7, 13, and 18 mm, respectively. In addition, the FCZ and FRC combination yielded significantly clearer and larger zones than the zones of either drug alone on the plain agar plate (Figure 1(c)). The sizes of the inhibition zone increased to 7 and 14 mm around the disks impregnated with 10 *μ*g of FCZ plus 625 *μ*g and 1250 *μ*g of FRC, respectively.

**Figure 2:**
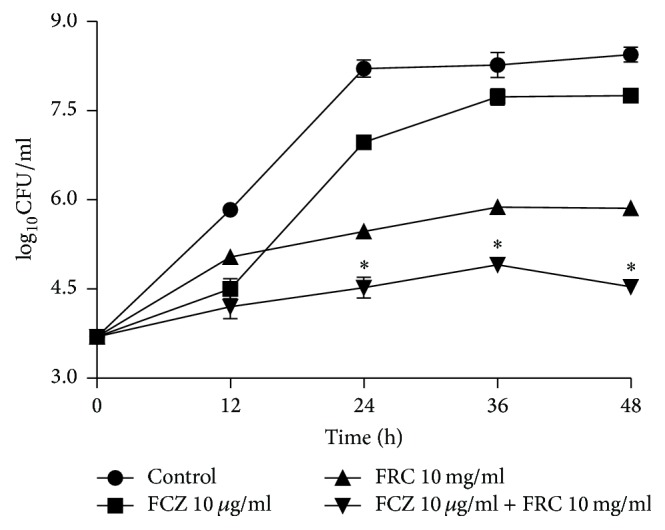
Time-kill curves of* C. albicans* isolate 103 (a clinical FCZ-resistant isolate) obtained using an initial inoculum of 10^3^ CFU/ml. The isolate was exposed to 10 *μ*g/ml FCZ or 10 mg/ml FRC alone, or in combination. CFUs were determined after 0, 12, 24, 36, and 48 h of incubation. The mean values for log_10 _CFU/ml versus time in three separate experiments are shown in the plots. ^*∗*^Synergism, log_10_ CFU/ml ≥ 2 antifungal activity produced by the combination compared with that of FCZ alone.

To confirm the synergism, the interaction was also detected with a time-kill curve assay (Figure 2). The results indicate that 10 *μ*g/ml FCZ alone exerted a weak antifungal effect, but 10 mg/ml FRC alone demonstrated a better antifungal effect after 24 h. More specifically, the antifungal effect of FCZ was improved by the addition of FRC. Given the initial inoculum of 10^3^ CFU/ml, the combination of FCZ and FRC caused a 2.25, 2.65, and 3.05-log_10 _CFU/ml decrease compared with 10 *μ*g/ml FCZ alone 24 h, 36 h, and 48 h later (Table 5). The synergistic antifungal effect was produced at 24 hours and the effect was stronger at 48 hours. These results suggested that FRC exerted a major time-dependent antifungal effect on the synergism of FCZ and FRC.

### 3.2. FRC and FCZ Combination Increases the Lifespan in a Mouse Model of Systemic Candidiasis

**Figure 3:**
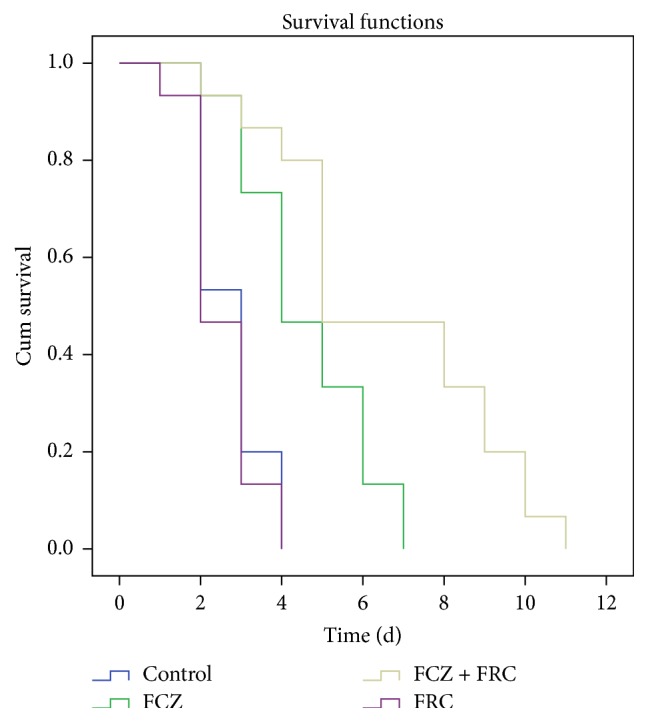
Effects of the combination of FRC and FCZ against systemic infection caused by* C. albicans* 103 in mice.

**Figure 4:**
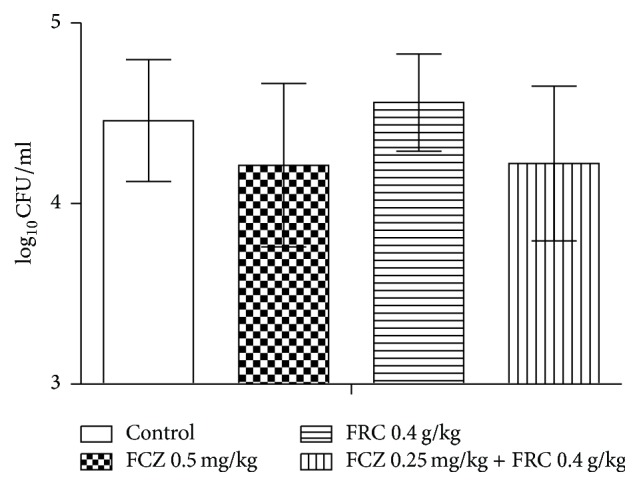
Fungal burdens in the kidneys of mice infected with* C. albicans* 103.

FRC showed antifungal activity in vitro; therefore, we examined this activity in vivo. For this purpose, a mouse model of systemic fungal infection was established. All mice were infected with the resistant* C. albicans* isolate 103 and were orally administered drugs. Four days later, all mice died in the control and FRC groups. Thus, administration of* C. albicans* 103 to the other two groups was stopped. Twelve days later, the other two groups of mice all died. We observed that the combination of FRC and FCZ increased the lifespan of the infected mice, indicating that this combination is protective during infection of a live animal. Survival analysis was performed using SPSS 17.0 (Figure 3). FRC was ineffective against* C. albicans* 103 (*P* > 0.05 versus control), while FCZ could prolong the survival time of the mice (*P* < 0.05 versus control). In addition, the combination of FRC and FCZ increased the lifespan of the infected mice, indicating that this combination is protective during the infection of live mice. The increased lifespan in the combination group was statistically significant as confirmed by Kaplan-Meier statistical analysis (*P* < 0.05). Therefore, consistent with its in vitro activities, FRC is an antifungal agent in this mouse model of systemic fungal infection.  Figure 4 presents the fungal burdens in the mouse kidneys. The combination of FCZ (0.25 mg/kg) and FRC (0.2 g/kg) reduced the number of CFU/g in the kidneys of mice infected by* C. albicans* 103, but there was no difference between the combination and FCZ alone groups. FRC at the dose of 0.4 g/kg daily was ineffective.

### 3.3. FRC Low Toxicity to Cells

The cytotoxic activities of FRC and FCZ were analyzed on a HUVEC cell line and showed LD_50_ at a concentration of 320 *μ*g/ml and 64 *μ*g/ml for FRC and FCZ, respectively. According to the checkerboard results, the MIC_80_ values for FRC were much lower than the LD_50_. When FRC was combined with FCZ, the LD_50_ of FCZ decreased from 64 *μ*g/ml to 4 *μ*g/ml (Table 6). FRC exerts a low toxicity to cells and can reduce the cytotoxic dose of FCZ.

### 3.4. FRC Decreases Content of Ergosterol

Given that synergistic antifungal activity was demonstrated in the studies described above, we then investigated the synergistic mechanism of FCZ and FRC against* C. albicans.* The sterol profile was studied by GC-MS using strain 103. As summarized in Table 7, ergosterol was the major sterol in strain 103, which accounted for 87.80% of the total sterols extracted from the control group. However, the ergosterol contents decreased to 8.93%, 8.38%, 1.81%, 1.72%, and 21.75% in the groups administered 1 *μ*g/ml FCZ, 1 *μ*g/ml FCZ + 25 *μ*g/ml FRC, 1 *μ*g/ml FCZ + 250 *μ*g/ml FRC, 1 *μ*g/ml FCZ + 2500 *μ*g/ml FRC, and 2500 *μ*g/ml FRC, respectively. The decreased contents of ergosterol were more significant when FCZ was combined with 250 *μ*g/ml or 2500 *μ*g/ml FRC. The consumption of ergosterol induced an increase in the eburicol fraction and a decrease in lanosterol. The sterol content changes were consistent with the FCZ inhibition activity of Erg11 in the ergosterol biosynthetic pathway.

## 4. Discussion

*C. albicans* is one of the most important opportunistic human fungal pathogens and causes diseases such as thrush and vaginitis [34]. Amphotericin B is widely used in the treatment of many systemic mycoses [35]. However, given their severe side effects, the azole drugs, especially FCZ, are widely used to fight* C. albicans* infections. Not surprising, repeated FCZ therapy for fungal infections in patients leads to emergence of serious FCZ-resistant* C. albicans*. Thus it is very important to identify new antifungal drugs.

FRC is included in the Chinese Pharmacopoeia for the treatment of menstrual disorders. For the first time, we have identified the antifungal capacity of FRC. In our work, the synergistic action of FCZ and FRC was shown in vitro and was confirmed by animal experiments in vivo. The results of FRC in combination with FCZ indicate its high biological activity. Moreover, FRC has low toxicity to the cell and can reduce the LD_50_ of FCZ in HUVEC cell line. The synergistic effect of FRC and FCZ against* C. albicans* in vitro and in vivo indicates that FRC is a promising Chinese herbal medicine for* C. albicans* resistant to FCZ.

In vitro experiments showed that the effective concentrations of FRC in different experiments were different. We found that as little as 20–40 *μ*g/ml can exert activity against* C. albicans* in the checkerboard test. However, up to 2500 *μ*g in the disk diffusion test was required to exert antifungal effects. However, in time-kill curve studies, the FRC can spread normally in a liquid environment and did not require high drug concentration to exhibit synergistic fungicidal activity. The agar diffusion test results were affected by drug diffusion. Agar and filter paper, which have network structures, can hinder macromolecular diffusion. Additionally, the FRC extract is a mixture. Considering these influencing factors, the diffusion capacity of FRC in agar cannot be equated to diffusion in liquid; therefore, the antifungal activity was significantly weaker than in the microdilution assay.

The main compounds in FRC are flavonoids, phenolic acid, ethereal oils, and tannin [36]. The flavonoids include kaempferol, kaempferol-3-O-a-L-arabinoside, kaempferol-3-O-a-L-rhamnoside, quercetin, and quercetin-3-O-a-L-arabinoside, in which kaempferol has antifungal effect [37]. It has also been reported that many phenolic compounds exert potent anti-*Candida* activities, and some of these compounds work synergistically or additively with FCZ [38–44]. We have simply separated different components from the hydroalcoholic extract. The checkerboard results showed that not only flavonoids exert the strongest antifungal effect, but also phenols. However, the antifungal effects of the separated components from the hydroalcoholic extract were weaker than that of the hydroalcoholic extract. We speculate that certain components of the extract of FRC hydroalcoholic have antifungal effect; all components mixed together can exert a stronger antifungal effect. Therefore more experiments must be performed to identify the specific effective ingredients.

The sterol profile analysis results show that FRC strengthens the FCZ-mediated inhibition of ergosterol biosynthesis. Ergosterol is an important sterol presented in the yeast cell membrane that controls membrane integrity and fluidity, and it is an important target for some antifungal drugs [45]. The antifungal mechanism of FCZ is inhibition of the activity of cytochrome P450 (Erg11p) responsible for the lanosterol 14*α*-demethylase (CYP51) to suppress ergosterol biosynthesis [46]. It can be seen from Table 7 that FRC can reduce the ergosterol synthesis, but the effect is weaker than FCZ. When FRC used with FCZ, the conversion of lanosterol to eburicol is significantly increased, leading to almost no synthesis of ergosterol. We conclude that FRC and FCZ get through different ways to change the normal metabolism direction of lanosterol: the effect of FCZ on the accumulation of 14*α*-methylsterols [47]. FCZ promotes the accumulation of 14*α*-methylsterols to reduce lanosterol; FRC reduces lanosterol by promoting the synthesis of eburicol. Both of them inhibit the synthesis of lanosterol to ergosterol. The synergistic effect is to further reduce the synthesis of ergosterol, interfering with the functions of ergosterol. Eventually, the fungus died due to cell membrane structure and function damage.

## 5. Conclusion

In this study we showed that FRC displayed the higher activity against FCZ-resistant clinical* C. albicans* and the lower cytotoxicity to cells when compared with azoles. The synergism showed the* Flos Rosae Chinensis* potential antifungal activity. As a traditional Chinese medicine, FRC antifungal activity suggests that we should find more effective substances for the treatment of fungal diseases and further explore the new use of traditional Chinese medicine.

## Figures and Tables

**Table 1:** Activity of FRC alone and in combination with FCZ against FCZ-resistant *C. albicans  *(*μ*g/ml)^a^.

*C. albicans*	Alone		Combination^b^		FIC index for combination^c^	Model of interaction
	FRC	FCZ	FRC	FCZ		
100	39.06	>64	4.88	0.125	0.126	syn
904	19.53	>64	4.88	0.125	0.25	syn
953	19.53	>64	4.88	0.125	0.25	syn
901	312.5	>64	2.44	1	0.016	syn
103	19.53	>64	2.44	0.125	0.126	syn
J5	19.53	>64	4.88	2	0.265	syn
32	19.53	>64	4.88	2	0.265	syn
842	19.53	>64	4.88	8	0.312	syn
557	19.53	>64	1.22	8	0.125	syn
J28	39.06	>64	4.88	1	0.133	syn
J38	39.06	>64	4.88	0.125	0.126	syn
112678#	19.53	>64	1.22	2	0.078	syn
502	39.06	>64	2.44	1	0.070	syn

^a^FCZ, fluconazole; FRC, *Flos Rosae Chinensis*; FIC index, fractional inhibitory concentration index; syn, synergism.

^b^MIC_80_s values for combinations are expressed as MIC_80_ of FCZ/MIC_80_ of FRC. High off-scale MIC_80_ values were converted to the next highest concentration.

^c^Synergy and antagonism were defined by FIC indices of <0.5 and >4, respectively. An FIC index result of >0.5 but <4 was considered indifferent.

**Table 2:** Activity of FRC alone and in combination with FCZ against FCZ-sensitive *C. albicans  *(*μ*g/ml)^a^.

*C. albicans*	Alone		Combination^b^		FIC index for combination^c^	Model of interaction
	FRC	FCZ	FRC	FCZ		
SC5314	19.53	1	2.44	1	1.125	indiff
AT10231	19.53	0.5	4.88	0.25	0.750	indiff
465	39.06	0.5	4.88	0.25	0.625	indiff
113187#	19.53	2	2.44	1	0.625	indiff
112721#	19.53	2	4.88	1	0.750	indiff

^a^FCZ, fluconazole; FRC, *Flos Rosae Chinensis*; FIC index, fractional inhibitory concentration index; indiff, indifference.

^b^MIC_80_s values for combinations are expressed as MIC_80_ of FCZ/MIC_80_ of FRC. High off-scale MIC_80_ values were converted to the next highest concentration.

^c^Synergy and antagonism were defined by FIC indices of <0.5 and >4, respectively. An FIC index result of >0.5 but <4 was considered indifferent.

**Table 3:** Activity of FRC alone and in combination with FCZ against other fungi (*μ*g/ml)^a^.

Fungi	Alone		Combination^b^		FIC index for combination^c^	Model of interaction
	FRC	FCZ	FRC	FCZ		
*C. krusei* 4996	19.53	64	4.88	>64	2.25	indiff
*C. tropicalis* 2718	78.125	64	4.88	0.25	0.066	indiff
*C. parapsilosis* 90018	4.88	0.5	2.44	0.25	1	indiff
*C. parapsilosis* 160	156.25	0.5	19.53	0.125	0.375	syn
*Aspergillus fumigatus* 7544	>10000	>64	156.25	>64	1.008	indiff
*M. gypseum*	625	32	19.53	0.125	0.035	syn
*T. rubrum*	312.5	16	19.53	0.125	0.07	syn

^a^FCZ, fluconazole; FRC, *Flos Rosae Chinensis*; FIC index, fractional inhibitory concentration index; syn, synergism; indiff, indifference.

^b^MIC_80_ values for combinations are expressed as MIC_80_ of FCZ/MIC_80_ of FRC. High off-scale MIC_80_ values were converted to the next highest concentration.

^c^Synergy and antagonism were defined by FIC indices of <0.5 and >4, respectively. An FIC index result of >0.5 but <4 was considered indifferent.

**Table 4:** Activity of FRC alone and in combination with other antifungal drugs against FCZ-resistant *C. albicans  *(*μ*g/ml)^a^.

Positive drug	Alone		Combination^b^		FIC index for combination^c^	Model of interaction	*C. albicans*
	FRC	Positive drug	FRC	Positive drug			
MCZ	39.06	64	2.44	0.125	0.064	syn	J5
MCZ	312.5	64	2.44	0.125	0.01	syn	901
MCZ	39.06	64	2.44	0.125	0.064	syn	103
KCZ	39.06	32	2.44	0.125	0.066	syn	J5
KCZ	312.5	32	2.44	0.125	0.012	syn	901
KCZ	39.06	32	2.44	0.125	0.066	syn	103
ICZ	39.06	>64	2.44	0.125	0.063	syn	J5
ICZ	312.5	>64	2.44	0.5	0.012	syn	901
ICZ	39.06	>64	2.44	0.25	0.064	syn	103
5-FLU	39.06	0.016	2.44	0.016	1.062	indiff	J5
5-FLU	312.5	0.25	39.06	0.125	0.625	indiff	901
5-FLU	19.53	0.25	1.22	0.25	1.062	indiff	103
AMB	39.06	0.5	4.88	0.5	1.125	indiff	J5
AMB	312.5	0.25	78.125	0.125	0.75	indiff	901
AMB	19.53	0.5	4.88	0.25	0.75	indiff	103

^a^KCZ, ketoconazole; ICZ, itraconazole; MCZ, miconazole; 5-Fc, 5-fluorocytosine; AMB, amphotericin B; FRC, *Flos Rosae Chinensis*; FIC index, fractional inhibitory concentration index; syn, synergism; indiff, indifference.

^b^MIC_80_ values for combinations are expressed as MIC_80_ of FCZ/MIC_80_ of FRC. High off-scale MIC_80_ values were converted to the next highest concentration.

^c^Synergy and antagonism were defined by FIC indices of <0.5 and >4, respectively. An FIC index result of >0.5 but <4 was considered indifferent.

**Table 5:** FRC combination with FCZ inhibited the growth of clinical isolate 103 of FCZ-resistant *C. albicans  *(CFU)^a^.

	Time (h)				
	0	12	24	36	48
Control					
avg	4966.667	676666.7	1.67*E* + 08	2*E* + 08	2.83*E* + 08
lg⁡(avg)	3.696065	5.830375	8.221849	8.301753	8.452298
SD	0.7409601	0.06247	0.5773503	0.867533187	0.578173
FCZ 10 *μ*g/mL					
average	4966.667	22133.33	6183333	37000000	39866667
lg⁡(avg)	3.696065	4.345047	6.791223	7.568202	7.60061
SD	0.74096	0.767858	0.654104	1.024609	0.890279
FRC 10 mg/mL					
average	4966.667	110400	293333.3	753333.3	720000
lg⁡(avg)	3.696065	5.042969	5.467361	5.876987	5.857332
SD	0.74096	0.422549	0.952448	0.54554	0.745681
FCZ 10 *μ*g/mL + FRC 10 mg/mL					
average	4966.667	17066.67	35000	81666.67	35166.67
lg⁡(avg)	3.696065	4.232149	4.544068^*∗*^	4.912045^*∗*^	4.546131^*∗*^
SD	0.74096	0.400816	0.795532	0.654104	0.957788

^a^FCZ, fluconazole; FRC, *Flos Rosae Chinensis*; avg, average. ^*∗*^Synergism, ≥2 log_10_ CFU/ml in antifungal activity produced by the combination compared with that of FCZ alone.

**Table 6:** Effects of FRC and FCZ on cell toxicity (*μ*g/ml).

	FCZ	FRC	FCZ + FRC
LD_50_	64	320	4/160

**Table 7:** Sterol compositions of *C. albicans* strain 103 following FCZ or FRC treatment, as analyzed by gas chromatography-mass spectrometry^a^.

	Control	FCZ 1 *µ*g/ml	FCZ 1 *µ*g/ml + FRC 25 *µ*g/ml	FCZ 1 *µ*g/ml + FRC 250 *µ*g/ml	FCZ 1 *µ*g/ml + FRC 2.5 mg/ml	FRC 2.5 mg/ml
Ergosterol	87.8	8.93	8.38	1.81	1.72	21.75
14a-Methyl-5a-ergosta-8,24(28)-dien-3a-ol	ND	3.2	2.87	ND	ND	ND
Ergosta-8,24(28)-dien-3-ol,4,14-dimethyl	1.76	6.45	27.12	2.36	2.02	8.78
Stigmast-5-en-3-ol	ND	ND	1.21	12.28	11.26	8.05
Eburicol	ND	55.54	55.9	70.83	72.93	56.03
Cholest-4-en-3-one	ND	ND	ND	0.7	0.34	ND

Lanosterol	ND	25.22	ND	9.05	9.99	ND
Ergosta-7,22-dien-3-ol	1.27	ND	ND	ND	ND	0.69
Ergosta-5,8-dien-3-ol	1.81	ND	ND	ND	ND	2.35
Cholest-7-en-3-one,4,4-dimethyl-	ND	3.2	ND	ND	ND	ND
Cholesta-8,24-dien-3-ol,(3á,5à)-(zymosterol)	1.26	ND	ND	ND	ND	ND
Ergosta-5,24(28)-dien-3-ol	1.12	0.65	3.97	2.07	1.14	0.86
Cholest-7-en-3-ol,4,4-dimethyl-	1.05	ND	ND	ND	ND	0.95
Unknown	3.93	ND	ND	ND	ND	ND
Ergost-5-en-3-ol	ND	ND	ND	0.9	0.6	0.55

^a^FCZ, fluconazole; FRC, *Flos Rosae Chinensis*; ND, not detected.
